# Internal Jugular Vein Blood Flow in Multiple Sclerosis Patients and Matched Controls

**DOI:** 10.1371/journal.pone.0092730

**Published:** 2014-03-27

**Authors:** Marcello Mancini, Roberta Lanzillo, Raffaele Liuzzi, Orlando Di Donato, Monica Ragucci, Serena Monti, Elena Salvatore, Vincenzo Brescia Morra, Marco Salvatore

**Affiliations:** 1 Institute of Biostructure and Bioimaging (IBB), Italian National Research Council (CNR), Naples, Italy; 2 IRCCS, SDN Foundation, Institute of Diagnostic and Nuclear Development, Naples, Italy; 3 Department of Neurosciences, Reproductive and Odontostomatological Sciences, Federico II University-School of Medicine, Naples, Italy; 4 Department of Advanced Biomedical Science, Federico II University-School of Medicine, Naples, Italy; INSERM U894, Centre de Psychiatrie et Neurosciences, Hopital Sainte-Anne and Université Paris 5, France

## Abstract

The aim of the study was to investigate the Internal Jugular Veins dynamics using contrast enhanced ultrasonography in Multiple Sclerosis patients, clinically isolated syndrome patients and healthy controls. Contrast enhanced ultrasonography imaging of the Internal Jugular Vein was performed in fifty-eight patients with Multiple Sclerosis, seven clinically isolated syndrome patients and in thirteen healthy controls. Time-intensity curves were quantified using a semi-automated method and compared with clinical disease outcomes. Wash-out parameters were calculated and six Time-intensity curves shapes were created. Significantly reduction of wash-out rate in Internal Jugular Veins was detected in Multiple Sclerosis patients compared to healthy controls [22.2% (2.7%–65.9%) vs. 33.4% (16.2%–76.8%); P<0.005]. Internal Jugular Vein enhancement was heterogeneous in patients with Multiple Sclerosis and consisted of slow wash-out Time-intensity curves shapes, compared with almost only one type of Time-intensity curves shape in control subjects that correspond to fast enhancement and fast wash-out. The vein wash-in parameters were similar in Multiple Sclerosis group compared with controls. A significant correlation was found between Internal Jugular Vein wash-out and level of disability (R = −0.402, p<0.05). Contrast enhanced ultrasonography of the Internal Jugular Vein with time intensity curve analysis revealed alterations of cerebral venous outflow in Multiple Sclerosis patients, however mechanisms that determine this condition remains unclear.

## Introduction

The most widely accepted hypothesis is that MS is an inflammatory and degenerative autoimmune disease that leads to destruction of CNS myelin, with multifactorial pathogenesis [1]. The early course of disease is characterized by episodes of neurological dysfunction that usually recover. However, over time the pathological changes become dominated by widespread microglial activation associated with extensive and chronic neurodegeneration, the clinical correlate of which is progressive accumulation of disability. Antimyelin T-cell-mediated inflammatory responses are believed to have a crucial role in the development of focal lesions [2], however, the underlying mechanism of the widespread axonal degeneration is not yet fully understood.

Abnormalities of cerebral hemodynamics in MS have been investigated by using single photon emission tomography, PET and perfusion MR imaging [3]–[5]. These studies have demonstrated widespread hypoperfusion in focal lesions, and in both the grey and white matter in patients with MS and all clinical phenotypes [4]–[5] and in the very early stage of the disease [6]. These findings are consistent with earlier histopathologic studies reporting vascular occlusive changes in MS, characterized by thrombosis of small veins and capillaries, vein wall hyalinization, and intravascular fibrin deposits [7]–[8]. Dawson described MS lesions as plaques mostly centered on small periventricular veins [9]. Recent studies, using high field MRI and susceptibility-weighted imaging have shown the perivascular inflammation pattern in MS by sensitively visualizing the small venous vessels in the periventricular and deep white matter [10]. Moreover MS patients showed a reduced visibility of cerebral veins that is inversely correlated with the periventricular and whole-brain T2-lesion load [11], low cerebral venous blood flow, low cerebral blood volume and higher MTT compared with healthy controls [12]–[13].

Compared with the cerebral arterial system, the cerebral venous system has been less well described and studied and this might lead to an underestimation of cerebral venous disorders. Unlike the carotid artery, the vascular wall of the IJV is much more flexible with a variable lumen diameter and this makes more difficult to perform a quantitative study of blood flow. The internal jugular veins (IJVs) are the main pathway for venous outflow in the supine position, while in the upright posture the IJVs collapse and venous outflow occurs mainly through secondary veins such as the vertebral, epidural, and deep cervical veins, which compose the vertebral venous plexus [14]–[15]. The hemodynamic of the IJVs may reflect cerebral venous drainage conditions and appear to influence intracranial hemodynamic, perfusion of the brain [16] and the dynamics of Cerebrospinal fluid system [17]. The insufficient cerebral venous drainage could cause a variety of unspecific central nervous symptoms such as headaches, visual disturbances, or amnestic dysfunctions [18]. Several studies have found a relationship between IJV drainage abnormalities and certain neurological diseases of undetermined aetiology [19]–[20] including leukoaraiosis [21] dementia [22] and normal-pressure hydrocephalus [17] and, recently, MS [23]–[24].

US contrast agents are true intravascular agents that, unlike the diffusible agents commonly used in MR imaging and CT, remain entirely within the vascular space and possess intravascular rheological characteristics similar to those of red blood cells [25]. Dynamic contrast–enhanced US imaging is the time-dependent registration of changes in ultrasonic signal intensity during and after intravenous injection of a contrast agent. The dependence of enhancement by time can be shown in a time-intensity curve (TIC) where the average signal from a Region of Interest (ROI) is plotted as a function of time. The integration of modern ultrasonographic equipment with dedicated signal-processing systems overcomes the operator-dependent analysis of the ultrasonic signal. From a TIC can be obtained some temporal and amplitude parameters, such as tissue and vascular uptake, transit times, and wash-out of the contrast agent.

The present study describes a new and simple approach to cerebral venous outflow analysis, based on the use of ultrasound contrast agent imaging with time intensity curve analysis. The analysis of IJV drainage patterns in HC and MS patients in horizontal body position was presented.

## Materials and Methods

### Patients

This study was approved by the institutional ethics committee for biomedical activities "Carlo Romano" Medical School University Federico II, Naples - Italy, and informed written consent was obtained from all subjects. Fifty-eight consecutive patients affected by clinically defined MS diagnosed according to the revised McDonald criteria [26], seven patients with Clinically Isolated Syndrome (CIS), and thirteen age- and sex-matched healthy controls were enrolled into the study.

Clinical assessment was done on the same week of the ultrasound study, and disability was graded by using Kurtzke's Expanded Disability Status Scale (EDSS) [27] and Multiple Sclerosis Severity Score (MSSS) [28].

Inclusion criteria for patients with MS were age 18–60 years and an EDSS between 0 and 6.5. Exclusion criteria were presence of relapse and steroid treatment in the 30 days preceding study entry for all patients, pregnancy or preexisting medical conditions known to be associated with brain pathology. ECG was performed and fasting venous blood sample was drawn for routine laboratory examination including blood clotting and screening for thrombophilic status.

### Ultrasound studies

Extracranial Duplex sonography was performed with Philips iU22 ultrasound instrument (*Bothell WA, U.S.A.*) with a 9–3 MHz, linear-array probe, to exclude carotid artery and/or IJV stenosis.

For contrast-enhanced perfusion imaging, the following settings were used: mechanical index, 0.06; acoustic output, 90%; gray map, one; and frame rate, 13 Hz. An area of the neck where the IJV and thyroid gland appeared in the same transverse plane was located by using color Doppler and designated for analysis. After 10 minutes with the subject at rest in supine position, a bolus of 2.4 mL of an echo-contrast enhancer (SonoVue; Bracco, Italy) was manually injected into the left antecubital vein using a 22-gauge catheter at 2 mL/sec. The injection was followed immediately by a 10-mL saline bolus injected at 5 mL/sec. A 1-minute B-mode cine loop was then acquired. Then, after 10 minutes, a second intravenous bolus of 2.4 mL of SonoVue was repeated for the evaluation of contralateral side. Electrocardiography was used to monitor the heart rate during the test.

A trained sonographer (M.M.) with more than 20 years of ultrasound experience performed all ultrasonographic assessments. The length of exam was 1 hour. The time intensity curve was analyzed offline in a blinded way, by using a time-signal intensity curve analysis program QLab (Philips Healthcare) that displayed the acoustic intensity (in decibels) during the acquisition time in a manually defined region of interest.

To reduce the artifacts caused by peak intensity variations, contrast agent arrival was defined as the time point of a stable signal intensity enhancement of at least 5 dB in the IJV.

Nine semiquantitative perfusion parameters were extracted from the time-intensity curves in the ROI designated for analysis at level of Internal Jugular Vein (IJV):

1. Arrival time was defined as the time from injection of contrast agent to the beginning of signal intensity increase;
2. Absolute Intensity Peak (dB) was the maximum intensity at the peak of the intensity curve;
3. Time to peak intensity (in seconds) was defined as the time from injection of contrast agent to reach the peak of contrast intensity;
4. Incremental Time (in seconds) was calculated as the difference between time to peak intensity and arrival time;
5. Enhancement slope (dB/s) was calculated as the ratio between intensity peak and incremental time
6. Area under the curve (AUC) during wash-in (dB x sec) was calculated from the point of the increase in image intensity greater than 5 dB above the baseline to the peak intensity;
7. AUC during washout (dBs) was calculated from the peak intensity to the end of 1 minute acquisition;
8. minute residue value (averaged contrast enhancement values at 58 to 60 seconds);
9. minute wash-out rate (calculated according to the following equation: 1-minute wash-out rate  =  (peak enhancement value – 1-minute residue value)/peak enhancement value ×100.

The IJV valve incompetence with retrograde flow was considered when microbubbles appeared on B-mode imaging in the internal Jugular vein before than in carotid artery.

#### Time Intensity Curve Shape Analysis

**Figure 1:**
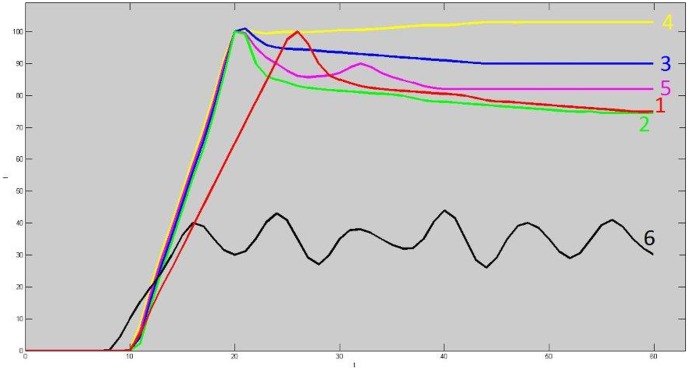
Graph of six time-intensity curves shape types (TIC). Type 1 shows slow enhancement followed by wash out phase; type 2 fast enhancement followed by wash out phase; type 3 fast enhancement followed by plateau phase; type 4 fast enhancement followed by gradual enhancement increase; type 5 fast enhancement followed by a short time wash out and by a second peak of enhancement; type 6 unclassified enhancement. Type 1–2 TIC shapes were considered indicative of fast wash-out. Type 3-4-5 were considered indicative of a slow venous wash-out.

The time-dependent signal intensity changes of imaged area was classified into one of six predefined TIC shape categories, which is associated with a color (Fig 1).

A curve was generated for every participant and the distribution of the different TIC shapes throughout the IJV was described (Fig.1).

#### Collateral Vein

We also assessed the left and right prominence of the other most important veins in the neck visible on CEUS imaging of the neck: external jugular veins, anterior jugular veins, facial veins, thyroid veins, and deep cervical veins. The number of collateral veins for the right and left side of the neck was counted.

### Statistical Analysis

Continuous data were reported as median and range, and categorical data as percentages. For descriptive statistics and analysis of continuous data the U test (Mann-Whitney) or, to compare more than two groups, Kruskal-Wallis analysis of variance was used. The post hoc analysis was performed by means of the Dunn test. The Spearman's rank correlation (ρ_s_) coefficient to assess inter-variable correlation was used. Fisher's exact test, and the χ^2^ test were used. Residual analysis was performed to assess differences in the distribution of venous criteria among MS subtypes. Significance was denoted when p was <0.05 by using two-tailed tests. Statistical analyses were performed using SPSS (version 18.0 Chicago, IL).

## Results

Demographic and clinical data of MS patients, CIS, and control groups are shown in Table 1. Significant differences in age, EDSS, MSSS and disease duration were evident in the subgroups. MS patients belonged to three clinical subtypes (32 RR, 20 SP and 6 PP), and were treated in 55% of cases with various disease modify therapies (interferon beta-1, natalizumab, glatiramer acetate, fingolimod, azathioprine, and mitoxantrone).

**Table 1:** Demographic and clinical characteristics of HCs, CIS patients, all MS patients and MS subtypes patients.

	HCs	CIS	MS Patients					
					MS Subtypes			
			Whole Group	p^§^	RR	SP	PP	p^*^
No.of Subjects	13	7	58		32	20	6	
M	9	3	13		7	2	4	
F	4	4	45	0.089	25	18	2	
Age (y)	35	33	41	0.002	35	43	49	0.001
	(23–54)	(27–41)	(25–60)		(25–54)	(27–55)	(35–60)	
Onset age(y)		27	29	0.336	26	31	36	0.061
		(24–41)	(8–47)		(8–37)	(19–37)	(22–47)	
Disease duration (y)		1	4	<0,001	8	11	15	0.002
		(0–7)	(2–7)		(1–31)	(2–30)	(6–18)	
EDSS		2	4.2	<0,001	3.5	4.5	6	<0,001
		(1,5–3,5)	(2–6,5)		(2,0–4,5)	(3,0–6,0)	(4,5–6,5)	
MSSS		2	1.78	<0,001	3.5	4.5	5.5	<0,001
		(1,5–3,5)	(0,13–12,76)		(2,0–4,5)	(4,0–6,0)	(4,5–6,5)	
								
								

Data are expressed as median and range (in brackets). There were no statistically significant differences between HCs and MS patients in age (p = 0.721; 0.153) or sex (p = 0.148; 0.332). § Mann-Whitney *U* test between CIS and MS patients. * Kruskal Wallis one-way analysis of variance among MS patients.

HCs =  healthy controls subjects; CIS  =  clinically isolated syndrome; MS =  multiple sclerosis; MS Subtypes: RR =  relapse remitting; SP =  secondary progressive, PP =  primary progressive.

The contrast agent was well tolerated in all individuals, without substantial side effects. Internal jugular vein wash-out rate and wash-out AUC were significantly increased in patients with MS compared with CIS and HCs (Table 2). Internal jugular vein reflux was observed in 14 veins. Even after jugular veins with retrograde flow were excluded from the statistical analysis, wash-out rate values remained significantly lower in the MS group compared to HCs and CIS [in MS patients without retrograde flow WO rate was 24.7% (range, 2.7–64.1%) CIS 29.2% (range, 13.3–42.9%) and HCs 35.4% (range, 16.2–76.8%) p<0.05]. No significant differences were found between the MS, CIS and HCs in all wash-in parameters excluding intensity peak and AUC wash-in (Table 3). The right jugular vein was significantly larger than left jugular vein (CSA in right IJV 0.83 cm^2^ vs. left IJV 0.67 cm^2^ p = 0.028) and showed lower WO rate [WO rate right 19.7% (2.74–53.44) left 24.9% (5.32–65.86)] and higher intensity peak [right 91.2 dB (5.88–122.68) left 87.13 dB (6.69–121.9)]. All wash-out time parameters were similar in male and female patients with MS. No difference was found between treated and untreated patients with MS (p = 0.857).

**Table 2:** Parameters of time intensity curve in Healthy Controls (HCs). CIS and patients with MS.

	HCs	CIS	MS patients					
						MS Subtypes		
			Whole group	*p*	RR	SP	PP	*p* *
IJV CSA supine (cm^2^)	0.8	0.4	0.8	*0.207*	0.7	0.8	1.2	*0.184*
	(0.4–2.1)	(0.3–1.3)	(0.1–3.4)		(0.1–2.2)	(0.1–3.4)	(0.1–2.3)	
*Wash*–*in parameters*								
Intensity Peak (dB)	81.2	83.2	91.0	*0.040*	88.6	92.7	102.6	*0.135*
	(4.9–99.6)	(53.0–121.5)	(5.9–122.7)		(5.9–122.7)	(7–121.9)	(10.5–119.7)	
Incremental Time (s)	9.0	9.5	10.1	*0.115*	10.1	10.0	10.4	*0.341*
	(6.9–14.1)	(5.9–12.9)	(4.9–27.9)		(5.1–27.9)	(4.9–18.3)	(5.5–17.1)	
Enhancement Slope (dB/s)	8.7	10.1	8.6	*0.534*	8.8	8.0	8.7	*0.784*
	(0.7–12.6)	(5.1–15.9)	(0.6–21.0)		(0.6–18.5)	(1.4–13.7)	(1.7–21.0)	
Time to Peak intensity (s)	23.3	22.0	23.2	*0.257*	23.2	22.6	26.0	*0.326*
	(18.1–33.4)	(16.2–26.2)	(6.5–38.0)		(14.8–38.0)	(6.5–36.6)	(18.4–33.6)	
AUC wash in (dBs)	346	442	505	*0.013*	501.5	527.8	741	*0.046*
	(14–715)	(260–705)	(19–1725)		(29–1725)	(19–1166)	(26–957)	
*Wash-out parameters*								
Residual value (dB)	53.9	57.2	70.4	*0.009*	66.8	72.0	85.5	*0.035*
	(1.5–72.1)	(34.8–98.4)	(70.4–114.2)		(3.7–114.2)	(4.3–101.3)	(4.3–105.0)	
Wash-out rate (%)	33.4	30.0	22.2	*0.003*	22.9	23.5	16.0	*0.014*
	(16.2–76.8)	(8.1–43.0)	(2.7–65.9)		(2.7–65.9)	(5.7–57.1)	(10.4–64.1)	
*Lower value*	28.3	21.6	17.1	*0.014*	18.2	18.2	14.5	*0.036*
	(16.2–45.1)	(8.6–36.1)	(2.7–49.5)		(2.7–49.5)	(5.7–46.8)	(10.4–24.5)	
AUC wash-out (dBs)	2 241	2 617	2 701	*0.023*	2 701	2 630	3 136	*0.098*
	(78–3 010)	(1 437–4 167)	(109–4 537)		(109–4 537)	(266–4 424)	(246–3 938)	

* Kruskal-Wallis analysis of variance.

All results are expressed for veins only the lower wash-out rate was evaluated for patients and calculated as the lowest value between the IJV for each patient.

**Table 3:** Cross-correlation matrix coefficients.

	Age (y)	Age at onset (y)	disease duration (y)	EDSS	MSSS	total relapses (n)	relapses in the last 2 years
IJV CSA (cm^2^)	0.215	0.155	0.195	0.089	0.300*	−0.061	0.105
*Wash-in parameters*							
Intensity Peak (dB)	0.129	0.238	−0.117	0.054	−0.069	0.207	−0.125
Incremental Time (s)	0.185	0.099	0.156	0.025	−0.089	−0.251	0.055
Enhancement Slope (dB/s)	−0.027	0.187	0.256*	−0.074	−0.115	0.247	−0.160
Time to Peak intensity (s)	0.286*	0.302*	0.124	0.012	0.032	−0.177	0.194
AUC wash in (dBs)	0.126	0.139	0.085	0.161	−0.061	0.025	−0.085
*Wash-out parameters*							
Wash-out rate (%)	−0.067	−0.204	−0.005	−0.038	0.044	−0.137	0.065
*Lower value*	−0.135	−0.346*	−0.082	−0.402*	−0.252	−0.120	0.024
AUC wash-out (dBs)	−0.019	0.179	−0.135	0.066	−0.118	0.222	−0.132

The numbers represents the Spearman's rank correlation coefficient value. * p<0.05.

There was a significant association between age at onset, increased EDSS and decreased WO rate in MS patients (table 3) calculated for each patient as the lowest value between the two IJV that is the value that takes into account for each patient the more diseased vein (age at onset r = −0346 EDSS r = −0.402, p<0.05, Table 3).

**Figure 2:**
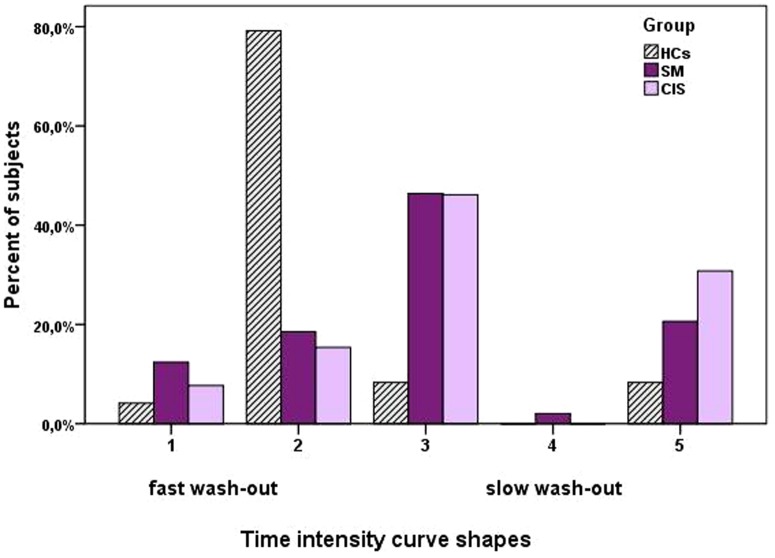
Frequency of Time intensity curve shapes in the different groups of subjects. The curve shapes 1-2 corresponds to a fast wash-out. The curve shapes 3-5 corresponds to slow wash-out.

The TIC pattern of the HCs consisted predominantly type 2 fast WO (fast wash-in enhancement followed by fast WO phase) and was observed in 73% of HCs in 16% of MS patients. The TIC pattern predominantly in MS patient was type 3 [fast wash-in enhancement followed by delayed WO (46.4% of MS patients and 8.3% of HCs; p<0.001)] (Figure 2).

Subjects with higher number of collateral veins (>2 collaterals) showed higher IJV intensity peak [82.3 dB (4.8–122.6) vs. 91.0 dB (5.9–122.7) p = 0.044]. A non-significant trend to higher residual value and AUC WO [residual value 59.2 dB (1.5–109.2) vs. 72.3 dB (3.7–114.2) p = 0.07; AUC WO (2 429 (78–4 464) vs. 2 757 (109–4 537) p = 0.083] was observed in MS patients with higher number of collaterals.

## Discussion

We utilized CEUS to describe and quantify the IJV outflow in MS, CIS and HC. The main result was that MS patients showed, on average, approximately 30% reduction of the venous outflow through the IJV in supine position measured as WO rate percentage. The WO rate was reduced more in patients with primary progressive MS than in patients with RR MS or CIS. These findings suggest than the altered IJV dynamics could be a consequence rather than a cause of MS.

These data are in agreement with other studies that showed impaired blood flow from the brain to the heart in patients with MS, with on average 63.5% higher hydraulic resistance of the cerebral-venous drainage system [29].

Altered venous outflow was also demonstrated in other studies using MR imaging [30], Pletismography [31] and Flebography [32] in patients with Multiple Sclerosis associated with CCSVI, a condition characterized by impaired venous outflow from the central nervous system to the heart [33]–[34]. One common feature of CCSVI is stenosis of one or both IJV with a greater cerebral venous outflow resistance [31] that could reduce the flow rate of the ultrasound contrast agent through the IJV.

The IJVs are the main drainage route for the cerebral venous system in supine position; in standing position these veins collapse and allow little blood flow. At steady-state the arterial inflow into the cranium must equal the venous outflow from the cranium. In MS patients the arterial inflow is preserved [35] since a reduction of venous outflow should result in an increase in the conduit's compliance, reduced flow pulsatility and increase outflow through collateral veins. Instead the upstream distention of the periventricular veins [36]–[37] is much more likely to be caused by constriction of the intracranial veins, perhaps by compression by inflammatory perivascular cuff.

The association of IJV WO rate with EDSS score suggest that outflow changes could be related to MS disability and pathophysiology. Although many studies showed higher frequency of IJVs anomalies in MS patients, there are not evidence for significant associations with disability [38]. Our results are comparable to the studies that utilize quantitative methodology to evaluate alteration of cerebral venous drainage [39]–[40]. The IJV wash-out rate could represent a more comprehensive quantitative measure of the severity of drainage impairment and, therefore, may be more useful in predicting clinical outcome than other imaging modalities that outcome categorical diagnosis.

The slope enhancement, peak intensity, and time to peak values are similar in MS and HCs and indicates that total amount of contrast agent passing through the ROI is similar in MS and HC and that venous filling with blood coming from the brain occurs with normal velocity. Therefore, the differences in the time courses (expressed as higher plateau of the wash-out phase) indicate an obstructive pattern with slower drainage of the vein, reduction of outflow and accumulation of contrast in the vessel.

Time Intensity Curve reflects the inflow and outflow venous dynamics represents venous physiology and could be associated with venous patency or obstruction. Using TIC shape analysis in our study, it was possible to demonstrate a significant difference between patients with MS and HCs. The TIC shapes differed significantly in the two groups. The type 2 TIC shape consists of a rapid enhancement phase followed by an early washout is the more frequently observed in healthy controls and is the normal IJV dynamics. TIC shapes with initial rapid enhancement followed by a plateau phase or gradual increase of the enhancement, or by a second peak of enhancement were associated with MS. The association of IJV TIC shapes and disease status is quite new in MS research and we believe that this simplified method might be useful to distinguish MS patients with or without associated vein outflow reduction/obstruction.

Whereas arterial flow is tightly regulated and primarily controlled by metabolic demand, the lower blood pressure of the venous system makes venous flow more dependent on physiological conditions such as respiration and posture. The motor mechanisms of cerebral venous return are represented by the cardiac output, the so called *vis a tergo*, and by aspiration of the atrium and pleural cavity, or *vis a fronte* [14]–[15]. The aspiration effect of the blood is a consequence of the negative pressure of the pleural cavity in inspiration [14]. Such mechanism of aspiration determined by the respiratory pump is particularly relevant in IJV. Therefore one hypothesis is that alteration of IJV outflow might be secondary to reduction of driving force of thoracic pump due to a weak respiratory muscular contraction of the chest [41] occurring early in the disease course and gradually worsening over time or secondary to some medications, such as muscle relaxants, that can depress breathing.

A raised pressure in the jugular veins could also be an earliest evidence of general systemic congestion, heart failure, hyperkinetic circulatory states, increased blood volume, bradycardia, increased intrapericardial, intrathoracic or intra-abdominal pressure, partial obstruction of the superior vena cava and tricuspid stenosis. However all these conditions have been ruled out in patients in this study who were almost all young and without other cardiac or systemic diseases associated with Multiple Sclerosis.

Moreover, compression of the veins draining the central nervous system by a muscle can be another cause of compromised cerebral venous outflow. The omohyoid muscle is usually located next to the IJV and it is known that an atypical omohyoid muscle can compress the IJV and in this way may affect cerebral venous drainage [42]. Recently Dolic et al., using MR venography, found that 22% of MS patients present with extraluminal abnormalities of the IJVs [43]. Jayaraman et al., who evaluated these veins using CT angiography [44], found a similar prevalence of severe extrinsic stenosis of the IJVs. Many investigators found reduced CBF in the GM and WM of patients with MS [2], [45], in the deep grey matter the magnitude of CBF reduction increase with the severity of the disease [46] suggesting a continuum of decreased tissue perfusion, beginning in the withe matter and spreading to the grey matter as the disease progresses.

Increased blood concentrations of the vasocostrictive compound endothelin-1 (ET-1) in patients with MS might also contribute to the slowdown of cerebral outflow [47].

A limit of the study is the low number of the control group due to ethical limitations to the use of contrast agent in normal subjects without any clinical indications.

A general limitation of quantifying replenishment kinetics is the dependence on the microbubble destruction and detection that could cause more complex replenishment kinetics.

Although the ultrasound contrast agent used in this study is known to be not soluble in blood and the low mechanical index harmonic imaging technique was used to limit bubble destruction, some microbubbles may have been inherently destroyed during the 1-minute scanning that we have used. Therefore, the wash-out of microbubbles in the region of interest on contrast-enhanced sonography might have been exaggerated compared with the actual blood wash-out and with the contrast wash-out in CT or magnetic resonance perfusion studies.

In conclusion, these results suggest that MS is associated with changes in the dynamics of the internal jugular vein.

## References

[1] Frohman EM, Racke MK, Raine CS (2006) Multiple sclerosis — the plaque and its pathogenesis. N Engl J Med 354: 942–955. 16510748 10.1056/NEJMra052130

[2] Compston A, Coles A (2008) Multiple sclerosis. Lancet 372: 1502–1517. 18970977 10.1016/S0140-6736(08)61620-7

[3] Sun X, Tanaka M, Kondo S, Okamoto K, Hirai S (1998) Clinical significance of reduced cerebral metabolism in multiple sclerosis: a combined PET and MRI study. Ann Nucl Med 12: 89–94. 9637279 10.1007/BF03164835

[4] Law M, Saindane AM, Ge Y, Babb JS, Johnson G, et al (2004) Microvascular abnormality in relapsing remitting multiple sclerosis: perfusion MR imaging findings in normal-appearing white matter. Radiology 231: 645–652. 15163806 10.1148/radiol.2313030996

[5] Inglese M, Park SJ, Johnson G, Babb JS, Miles L, et al (2007) Deep gray matter perfusion in multiple sclerosis: dynamic susceptibility contrast perfusion magnetic resonance imaging at 3 T. Arch Neurol. 64: 196–202. 10.1001/archneur.64.2.196 17296835

[6] Varga AW, Johnson G, Babb JS, Herbert J, Grossman RI, et al (2009) White matter hemodynamic abnormalities precede sub-cortical gray matter changes in multiple sclerosis. J Neurol Sci 282: 28–33. 19181347 10.1016/j.jns.2008.12.036 PMC2737614

[7] Wakefield AJ, More LJ, Difford J, McLaughlin JE (1994) Immunohistochemical study of vascular injury in acute multiple sclerosis. J Clin Pathol 47: 129–133. 8132826 10.1136/jcp.47.2.129 PMC501826

[8] Allen IV (1981) The pathology of multiple sclerosis-fact, fiction and hypothesis. Neuropathol Appl Neurobiol 7: 169–182. 7242846 10.1111/j.1365-2990.1981.tb00087.x

[9] Dawson JD (1916) The histology of disseminated sclerosis. Transactions of The royal Society of Edinburgh 50: 517–740.

[10] Kilsdonk ID, de Graaf WL, Barkhof F, Wattjes MP (2012) Inflammation high-field magnetic resonance imaging. Neuroimaging Clin N Am 22: 135–157. 22548925 10.1016/j.nic.2012.02.010

[11] Sinnecker T, Bozin I, Dörr J, Pfueller CF, Harms L, et al (2013) Periventricular venous density in multiple sclerosis is inversely associated with T2 lesion count: a 7 Tesla MRI study. Mult Scler 19(3): 316–25. 22736752 10.1177/1352458512451941

[12] Garaci FG, Marziali S, MeschiniA, Fornari M, Rossi S, et al (2012) Brain hemodynamic changes associated with chronic cerebrospinal venous insufficiency are not specific to multiple sclerosis and do not increase its severity. Radiology 265: 233–9. 22915599 10.1148/radiol.12112245

[13] Ge Y, Zhang Z, Lu H, Tang L, Jaggi H, et al (2012) Characterizing brain oxygen metabolism in patients with multiple sclerosis with T2-relaxation-under-spin-tagging MRI. J Cereb Blood Flow Metab 32: 403–412. 22252237 10.1038/jcbfm.2011.191 PMC3293125

[14] Schaller B (2004) Physiology of cerebral venous blood flow: from experimental data in animals to normal function in humans. Brain Res Rev 46: 243–260. 15571768 10.1016/j.brainresrev.2004.04.005

[15] Valdueza JM, von Munster T, Hoffman O, Schreiber SJ, Einhaupl KM (2000) Postural dependency of the cerebral venous outflow. Lancet 355: 200–201. 10.1016/s0140-6736(99)04804-7 10675123

[16] Frydrychowski AF, Winklewski PJ, Guminski W (2012) Influence of acute jugular vein compression on the cerebral blood flow velocity, pial artery pulsation and width of subarachnoid space in humans. PLoS One 7, e48245. 10.1371/journal.pone.0048245 PMC3480498 23110218

[17] Bateman GA (2002) Pulse-wave encephalopathy: a comparative study of the hydrodynamics of leukoaraiosis and normal-pressure hydrocephalus. Neuroradiology 44: 740–748. 12221445 10.1007/s00234-002-0812-0

[18] Digre KB (2002) Idiopathic intracranial hypertension headache. Curr Pain Headache Rep 6: 217–225. 12003693 10.1007/s11916-002-0038-1

[19] Doepp F, Bähr D, John M, Hoernig S, Valdueza JM (2008) Internal jugular vein valve incompetence in COPD and primary pulmonary hypertension. J Clin Ultrasound 36(8): 480–484. 18335510 10.1002/jcu.20470

[20] Doepp F, Valdueza JM, Schreiber SJ (2008) Incompetence of internal jugular valve in patients with primary exertional headache: a risk factor? Cephalalgia 28 (2): 182–185. 10.1111/j.1468-2982.2007.01484.x 18021266

[21] Chung CP, Hu HH (2010) Pathogenesis of leukoaraiosis: role of jugular venous reflux. Med Hypotheses 75: 85–90. 20172657 10.1016/j.mehy.2010.01.042

[22] Chung CP, Wang PN, Wu YH, Tsao YC, Sheng WY, et al (2011) More severe white matter changes in the elderly with jugular venous reflux Ann Neurol. 69: 553–559. 10.1002/ana.22276 21391231

[23] Zamboni P, Galeotti R, Menegatti E, Malagoni AM, Tacconi G, et al (2009) Chronic cerebrospinal venous insufficiency in patients with multiple sclerosis. J Neurol Neurosurg Psychiatry 80: 392–399. 19060024 10.1136/jnnp.2008.157164 PMC2647682

[24] Zivadinov R, Marr K, Cutter G, Ramanathan M, Benedict RH, et al (2011) Prevalence, sensitivity, and specificity of chronic cerebrospinal venous insufficiency in MS. Neurology 77: 138–144. 21490322 10.1212/WNL.0b013e318212a901

[25] Delorme S, Krix M (2006) Contrast-enhanced ultrasound for examining tumor biology. Cancer Imaging 6: 148–152. 17015239 10.1102/1470-7330.2006.0023 PMC1693765

[26] Polman CH, Reingold SC, Banwell B, Clanet M, Cohen JA, et al (2011) Diagnostic criteria for multiple sclerosis: 2010 Revisions to the McDonald criteria. Ann Neurol 69: 292–302. 21387374 10.1002/ana.22366 PMC3084507

[27] Kurtzke JF (1983) Rating neurologic impairment in multiple sclerosis: an expanded disability status scale (EDSS). Neurology 33(11): 1444–1452. 6685237 10.1212/wnl.33.11.1444

[28] Roxburgh RH, Seaman SR, Masterman T, Hensiek AE, Sawcer SJ, et al (2005) Multiple Sclerosis Severity Score Using disability and disease duration to rate disease severity Neurology. 64: 1144–1151. 10.1212/01.WNL.0000156155.19270.F8 15824338

[29] Zamboni P, Menegatti E, Conforti P, Shepherd S, Tessari M, et al (2012) Assessment of cerebral venous return by a novel plethysmography method. J Vasc Surg 56: 677–685. 22521804 10.1016/j.jvs.2012.01.074

[30] Haacke EM, Feng W, Utriainen D, Trifan G, Wu Z, et al (2012) Patients with multiple sclerosis with structural venous abnormalities on MR imaging exhibit an abnormal flow distribution of the internal jugular veins. J Vasc Interv Radiol. 23: 60–68. 10.1016/j.jvir.2011.09.027 22221473

[31] Beggs C, Shepherd S, Zamboni P (2013) Cerebral venous outflow resistance and interpretation of cervical plethysmography data with respect to the diagnosis of chronic cerebrospinal venous insufficiency *Phlebology, phleb.2012.012039, first published on May 3* 10.1258/phleb.2012.012039 23060482

[32] Veroux P, Giaquinta A, Perricone D, Lupo L, Gentile F, et al (2013) Internal jugular veins out flow in patients with multiple sclerosis:a catheter venography study. J Vasc Interv Radiol. 24(12): 1790–7. 10.1016/j.jvir.2013.08.024 24409471

[33] Zamboni P, Galeotti R, Menegatti E, Malagoni AM, Tacconi G, et al (2009) Chronic cerebrospinal venous insufficiency in patients with multiple sclerosis. J Neurol Neurosurg Psychiatry 80(4): 392–399. 19060024 10.1136/jnnp.2008.157164 PMC2647682

[34] Zamboni P, Menegatti E, Galeotti R, Malagoni AM, Tacconi G, et al (2009) The value of cerebral Doppler venous haemodynamics in the assessment of multiple sclerosis. J Neurol Sci 282(1-2): 21–27. 19144359 10.1016/j.jns.2008.11.027

[35] Mancini M, Morra VB, Di Donato O, Maglio V, Lanzillo R, et al (2012) Multiple sclerosis: cerebral circulation time. Radiology 262(3): 947–955. 22357894 10.1148/radiol.11111239

[36] Putnam TJ, Adler A (1937) Vascular architecture of the lesions of multiple sclerosis. Arch Neurol Psychiat 38: 1–5.

[37] Gaitán MI, de Alwis MP, Sati P, Nair G, Reich DS (2013) Multiple sclerosis shrinks intralesional, and enlarges extralesional, brain parenchymal veins. Neurology 80: 145–151. 10.1212/WNL.0b013e31827b916f PMC3589186 23255828

[38] Lanzillo R, Mancini M, Liuzzi R, Di Donato O, Salvatore E, et al. (2013). Chronic cerebrospinal venous insufficiency in Multiple Sclerosis: an highly prevalent age-dependent phenomenon. BMC Neurology, 13–20 doi:10.1186/1471-2377-13-20 10.1186/1471-2377-13-20 PMC3577443 23406210

[39] Denislic M, Milosevic Z, Zorc M, Ravnik IZ, Mendiz O (2013) Disability caused by multiple sclerosis is associated with the number of extracranial venous stenoses: possible improvement by venous angioplasty. Results of a prospective study. Phlebology 28: 353–360. 23202144 10.1258/phleb.2012.012065

[40] Weinstock-Guttman B, Ramanathan M, Marr K, Hojnack D, Benedict RH, et al (2012) Clinical correlates of chronic cerebrospinal venous insufficiency in multiple sclerosis. BMC Neurol 2012 12: 26. 10.1186/1471-2377-12-26 PMC3462121 22587224

[41] Gosselink R, Kovacs L, Decramer M (1999) Respiratory muscle involvement in multiple. Eur Respir J 13: 449–454. 10065697 10.1183/09031936.99.13244999

[42] Gianesini S, Menegatti E, Mascoli F, Salvi F, Bastianello S, et al. (2013) The omohyoid muscle entrapment of the internal jugular vein. A still unclear pathogenetic mechanism. Phlebology [Epub ahead of print] doi:10.1177/0268355513489549 10.1177/0268355513489549 23761870

[43] Dolic K, Marr K, Valnarov V, Dwyer MG, Carl E, et al (2012) Intra- and Extraluminal Structural and Functional Venous Anomalies in Multiple Sclerosis, as Evidenced by 2 Noninvasive Imaging Techniques. AJNR Am J Neuroradiol 33(1): 16–23. 22194367 10.3174/ajnr.A2877 PMC7966175

[44] Jayaraman MV, Boxerman JL, Davis LM, Haas RA, Rogg JM (2012) Incidence of extrinsic compression of the internal jugular vein in unselected patients undergoing CT angiography. AJNR Am J Neuroradiol 33(7): 1247–1250. 22322614 10.3174/ajnr.A2953 PMC7965500

[45] Ge Y, Law M, Johnson G, Herbert J, Babb JS, et al (2005) Dynamic susceptibility contrast perfusion MR imaging of multiple sclerosis lesions: characterizing hemodynamic impairment and inflammatory activity. AJNR Am J Neuroradiol 26: 1539–1547. 15956527 PMC8149080

[46] Inglese M, Adhya S, Johnson G, Babb JS, Miles L, et al (2008) Perfusion magnetic resonance imaging correlates of neuropsychological impairment in multiple sclerosis. J Cereb Blood Flow Metab 28: 164–171. 17473851 10.1038/sj.jcbfm.9600504 PMC2596621

[47] Haufschild T, Shaw SG, Kesselring J, Flammer J (2001) Increased endothelin-1 plasma levels in patients with multiple sclerosis. J Neuroophthalmol 21: 37–38. 11315981 10.1097/00041327-200103000-00011

